# Clinical features of gastric duplications: evidence from primary case reports and published data

**DOI:** 10.1186/s13023-021-01992-1

**Published:** 2021-08-19

**Authors:** Yang Li, Chen Li, Hao Wu, Quan Wang, Zhi-Dong Gao, Xiao-Dong Yang, Ke-Wei Jiang, Ying-Jiang Ye

**Affiliations:** 1 Department of Gastroenterological Surgery, Laboratory of Surgical Oncology, Beijing Key Laboratory of Colorectal Cancer Diagnosis and Treatment Research, Peking University People’s Hospital, No.11 Xizhimen South Street, Xicheng District, Beijing, 100044 China; 2 Department of Gastroenterological Surgery, Shandong Provincial Hospital, Cheeloo College of Medicine, Shandong University, Jinan, Shandong Province China

**Keywords:** Alimentary tract duplications, Gastric duplications, Evidence, Literature review

## Abstract

**Background:**

Alimentary tract duplications are rare congenital lesions, and only 2–8% of them are located in the stomach. Gastric duplications (GD) can lead to severe adverse events. Thus, surgical resection is required once the disease is diagnosed. The main purpose of this study is to describe the clinical features of gastric duplications and to provide evidence for the diagnosis and treatment.

**Methods:**

A retrospective review of eight gastric duplications at two medical centers Peking University People’s Hospital (PKUPH) and Shandong Provincial Hospital from 2010 to 2020 was conducted. Furthermore, the literature search was also conducted by retrieving data from PubMed, EMBASE and Cochrane Library databases from the date of the database inception to January 15, 2021.

**Results:**

Eight patients who were diagnosed as gastric duplications and 311 published records were included in this study. In all, 319 patients were identified: Vomiting and abdominal pain were the most frequent clinical presentations among juveniles and adults respectively. There was no difference in gender distribution (F: 53.16% vs M: 46.84%), and the cystic gastric duplications were the most common type of the gastric duplications (87.04%). More than half (53.30%) of included cases were located in the greater curvature of stomach.

**Conclusions:**

Gastric duplications could present with a wide spectrum of symptomatology, which might be misdiagnosed easily as other diseases. For cystic gastric duplications, the optimal treatment was a complete surgical removal. But conservative treatment might be an alternative strategy for tubular gastric duplications.

## Background

Alimentary tract duplications are uncommon congenital lesions which can occur anywhere from the mouth to the anus and have a reported incidence of approximately 1 out of 4500 births [1, 2]. Ileum is the most common site of this deformity, followed by esophagus, jejunum, colon, stomach and appendix [3–6]. Only 2–8% of all gastrointestinal tract duplications occurred in the stomach [7, 8]. Two forms of gastric duplications (GD) have been reported, namely, cystic and tubular. The cystic type composes around 80% of gastric duplications and they are not communicating with the gastric lumen. And the remaining 20% are the tubular GD, which are contiguous with the stomach and usually show some communication with the gastric lumen [9, 10].

The clinical symptoms and signs of GD can vary according to the age of the patients as well as the lesion’s sizes, locations and types [4]. The manifestations include abdominal pain, vomiting, hematemesis, weight loss and etc. However, some GD especially in adults may be totally asymptomatic, and might be identified on routine physical examination.

Although there are many auxiliary examinations such as computed tomography (CT), ultrasound, magnetic resonance imaging (MRI) and endoscopic ultrasonography (EUS), the preoperative diagnosis of GD is still very difficult due to the lack of specific symptoms and signs. GD are often misdiagnosed as gastrointestinal stromal tumor (GIST) or other gastric tumors [11, 12].

Currently, there has been limited analysis of GD’s clinical characteristics, etiology, pathologies, and the optimal treatment strategy. This study aims to describe the clinical features of GD in detail based upon the current patients’ data from a literature reviews, with the expectation to provide evidence in the diagnosis and treatment of GD.

## Methods

**Fig. 1:**
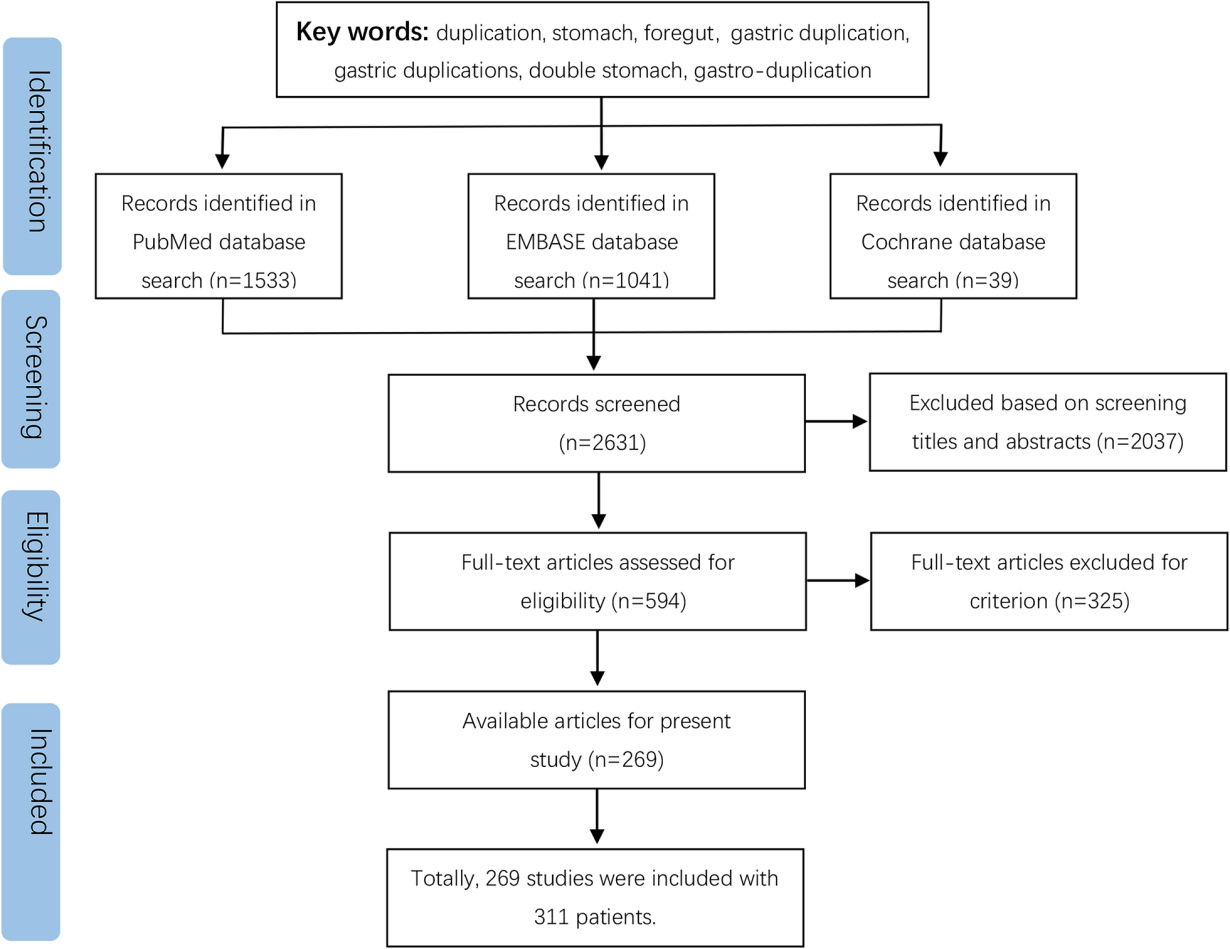
PRISMA flow chart of studies selection

To fully describe the demographic and clinical characteristics, we retrospectively collected all included patients’ data from Peking University People’s Hospital (PKUPH) and Shandong Provincial Hospital during 1st January, 2010 to 31st December, 2020. Patients’ information was extracted from the electronic medical records system, and follow-up were conducted via telephone conversations and outpatient visits. From 2010 to 2020, all patient records with a diagnosis of GD were reviewed after getting Ethical Committee approval. In addition, as shown in Fig. 1, we also searched the PubMed, EMBASE, and Cochrane Library databases. The search items were as follows: gastric duplications, double stomach, gastric duplication cyst, using Medical Subject Headings terms combined with free text terms. We also performed a supplementary literature search through Google Scholar.

The search strategies were determined by two researchers after several pre-searches. The search was completed on January 15, 2021. All article types (including case report, case series, etc.) would be included as long as they include the description of GD cases. Then we strictly screened the cases which reported in the articles, to ensure the integrity of the collected data. Only the cases would be included in our study when they reported at least four of following: gender, age, the location of GD, the size of GD, the type of GD, treatment strategy and follow-up information. Gastric duplications which separated of the stomach would be excluded in this retrospective study and review.

Patient demographics, the location of GD, patient symptoms, the investigation used in the diagnosis, the treatment provided, and the pathologic evaluation of the resected specimens were collected.

### Statistical analysis

Continuous variables were expressed as means and categorical variables were expressed as number (%). The statistical analyses were conducted using SPSS software (version 25.0; IBM Corp., Armonk, NY, USA), unless otherwise indicated.

## Results

A total of 319 patients diagnosed GD were included in our study, including eight patients (2.50%) from the two medical centers and 311 patients (97.50%) from previous published papers. The characteristics of these included patients were showed in Table 1.

**Table 1:** Clinical feature of patients with gastric duplications

	Literature-based patients (n = 311)	Two-centers patients (n = 8)	All included patients (n = 319)
Sex	Valid = 293	Valid = 8	Valid = 301
Male	139 (47.44%)	2 (2/8)	141 (46.84%)
Age (years)	Valid = 305	Valid = 8	Valid = 313
Range	0–82	1–55	0–82
Mean (SD)	20.77 (23.15)	30.63 (20.82)	21.02 (23.10)
Type	Valid = 239	Valid = 8	Valid = 247
Cystic	207 (86.61%)	8(8/8)	215 (87.04%)
Tubular	32 (13.39)	0(0/8)	32 (12.96%)
Sites	Valid = 264	Valid = 8	Valid = 272
Greater curvature	142 (53.79%)	3 (3/8)	145 (53.30%)
Lesser curvature	22 (8.33%)	3 (3/8)	25 (9.20%)
Cardia	18 (6.82%)	0 (0/8)	18 (6.60%)
Pylorus	47 (17.80%)	1 (1/8)	48 (17.65%)
AW	5 (1.89%)	1 (1/8)	6 (2.20%)
PW	30 (11.36%)	0 (0/8)	30 (11.00%)
Other anomalies	Valid = 85	Valid = 0	Valid = 85
Ectopic pancreas	33 (38.82%)	–	33 (38.82%)
Other sites duplications	14 (16.47%)	–	14 (16.47%)
Pulmonary sequestration	6 (7.06%)	–	6 (7.06%)
Congenital heart disease	6 (7.06%)	–	6 (7.06%)
Vertebral abnormalities	4 (4.71%)	–	4 (4.71%)
Other pancreas anomalies	12 (14.12%)	–	12 (14.12%)
Other anomalies	11 (12.94%)	–	11 (12.94%)
Presentation	Valid = 289	Valid = 8	Valid = 297
Symptomatic	228 (78.89%)	3 (3/8)	231 (77.78%)
Abdominal pain	105 (46.05%)	0 (0/3)	105 (45.45%)
Vomiting	105 (45.45%)	0 (0/3)	105 (45.45%)
Abdominal distension	17 (7.46%)	1 (1/3)	18 (7.79%)
Gastrointestinal hemorrhage	31 (13.59%)	0 (0/3)	31 (13.42%)
Fever	15 (6.58%)	0 (0/3)	15 (6.49%)
Weight loss	23 (10.09%)	0 (0/3)	23 (9.96%)
Others	19 (8.33%)	2 (2/3)	21 (9.09%)
Asymptomatic	61 (21.11%)	5 (5/8)	66 (22.22%)
Treatment strategy	Valid = 272	Valid = 8	Valid = 280
Surgery	256 (94.12%)	8 (8/8)	264 (94.29%)
Local resection (LR)	181 (70.70%)	6 (6/8)	187 (70.83%)
Partial gastrectomy (PG)	63 (24.61%)	2 (2/8)	65 (24.62%)
Radical resection (RR)	10 (3.91%)	0(0/8)	10 (3.79%)
LR + RR	2 (0.78%)	0(0/8)	2 (0.76%)
Conservative treatment	16 (5.88%)	0 (0/8)	16 (5.71%)
Follow-up			
Duration (month)	Valid = 75	Valid = 2	Valid = 77
Range	1–120	6–18	1–120
Mean (SD)	17.51 (19.44)	12 (8.49)	17.36 (19.23)
Outcomes	Valid = 75	Valid = 2	Valid = 77
Recurrence	1 (1.33%)	0 (0/2)	1 (1.30%)
Metastasis	5 (6.67%)	0 (0/2)	5 (6.49%)
ANED	61 (81.33%)	2 (2/2)	63 (81.82%)
AWD	3 (4.00%)	0 (0/2)	3 (3.90%)
DOD	5 (6.67%)	0 (0/2)	5 (6.49%)

### Analysis of primary cases

These eight patients were all cystic type of GD, and all patients underwent surgical resection. GD was correctly diagnosed preoperatively in only three patients, and three patients of all the five misdiagnosis patients were misdiagnosed as GIST. The mean size of the eight GD was 5.93 ± 4.26 cm and the median size was 4.6 cm. The largest size of GD was 15 cm, which was found in a 38-year-old female, who admitted to the hospital with abdominal distension. In our cases, the greater curvature and lesser curvature of stomach are the most common sites of GD, which were seen in three patients respectively. The other features were shown in Table 1.

### Pooling analysis of the all patients

As shown in Fig. 1, the initial search identified 2631 articles from PubMed, EMBASE and Cochrane Library databases, and after screening, a total of 269 articles were included finally. From Table 1, after data extraction of the 269 studies, 311 patients with GD were included at last.

**Fig. 2:**
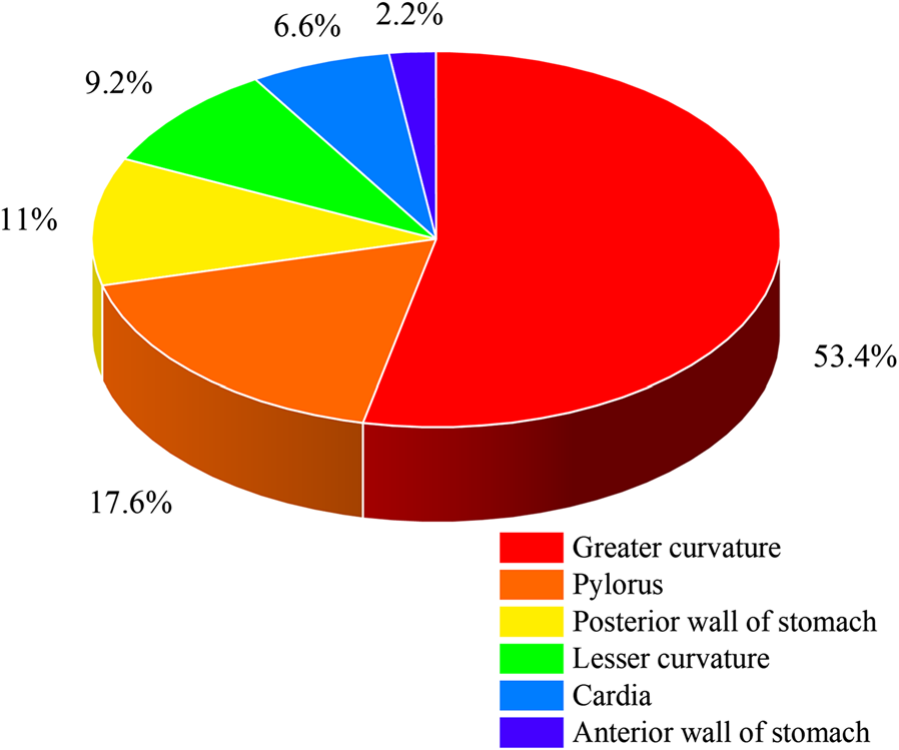
The location and frequency of gastric duplications

Of all these 319 patients (eight primary cases, 311 literature-based cases), 53.16% (160 out of 301) were female and 46.84% (141 out of 301) were male. The mean age of onset was 21.02 ± 23.10 years (range: 1 day to 82 years). Child patients accounted for 55.27% (173 out of 313). Nearly 38.65% (121 out of 313) of patients were under two years of age. The mean size of the GD was 5.79 ± 0.27 cm, and the median was 5.0 cm (range 0.6–40 cm). The cystic GD was the most common type of GD, accounting for 87.04% (215 out of 247) of all these patients. The location and frequency of GD are showed in Fig. 2. The greater curvature and pylorus were the most frequent sites of GD, harboring 53.30% (145 out of 272) and 17.65% (48 out of 272) of these lesions, respectively.

Geographically, more than half (71.43%, 225 out of 315) of these cases were reported from Asia (38.10%, 120 out of 315) and North America (33.33%, 105 out of 315). In addition, 20.95% (66 out of 315), 4.44%, (14 out of 315), 2.54% (8 out of 305), 0.63% (2 out of 315) from Europe, Africa, Australia and South America respectively. While, we analyzed that this difference may be related to the total population number and medical level in different regions.

**Fig. 3:**
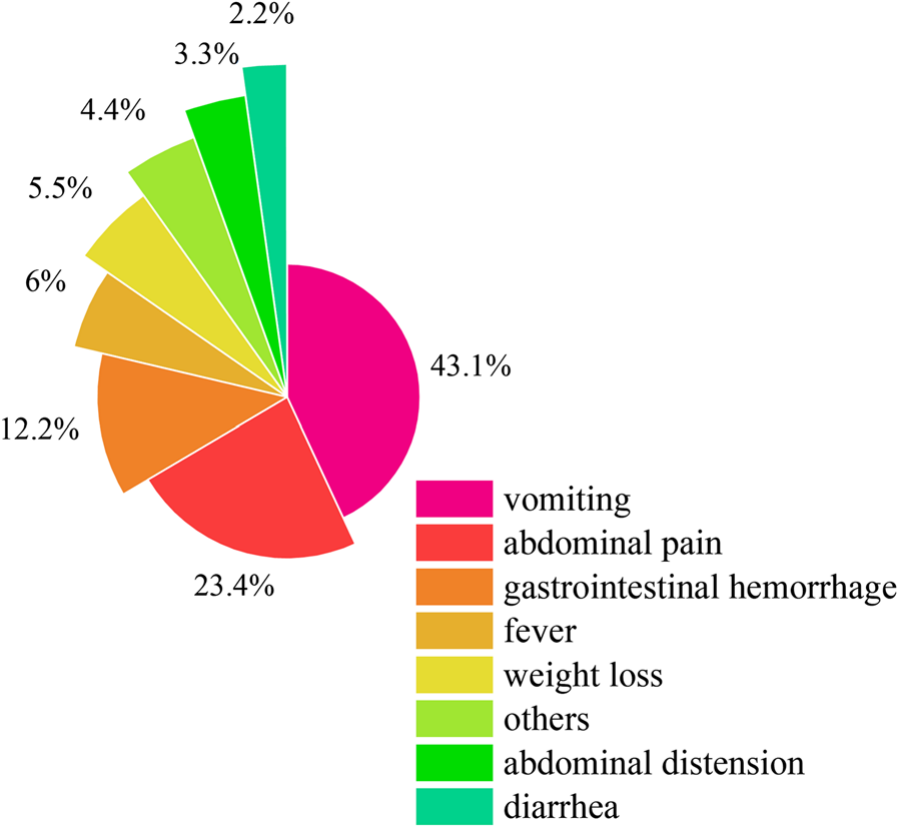
The presentation and constituent ratio of child patients

The clinical presentation of these lesions was polymorphous and it depended on the age of the patient and the size of the cyst. For child patients, vomiting was the most frequent association (61.20%, 79 out of 129), followed by abdominal pain (33.33%, 43 out of 129), gastrointestinal hemorrhage (16.28%, 21 out of 129), fever (8.53%, 11 out of 129), weight loss (7.75%, 10 out of 129), abdominal distension (4.65%, 6 out of 129) and diarrhea (3.10%, 4 out of 129). The constituent ratio of clinical presentation of child patients was shown in Fig. 3. While for adults, abdominal pain (61.38%, 62 out of 101) was the most common presentations, followed by vomiting (25.74%, 26 out of 101), weight loss (12.87%, 13 out of 101), abdominal distension (11.9%, 12 out of 101) and gastrointestinal hemorrhage (9.90%, 10 out of 101). In addition, 22.22% (66 out of 297) of all these patients were asymptomatic, and asymptomatic individuals were more common in adults (26.28%, 36 out of 137). However, among pediatric patients, only 18.87% (30 out of 159) were asymptomatic. There were 23.20% (74 out of 319) patients accompanying with other gastric diseases such as gastric ulcer and gastritis.

In this study, 26.6% of patients (85 out of 319) had other anomalies associated with GD. Among them, ectopic pancreas was the most common congenital malformation accounting for 38.82% (33 out of 85). 14 out of the 85 patients (16.47%) had a combination of duplication malformations at other sites, such as esophageal duplications, ureteral duplications and small intestinal duplications. In addition, only 4 patients (4.71%) had a vertebral anomaly.

The preoperative diagnosis of GD was difficult and a large proportion of the patients were misdiagnosed with other diseases. There were only 35.11% (112 out of 319) patients had a preoperative diagnosis suggestive of GD, especially in adults, with the rate of misdiagnosis as high as 72.14% (101 out of 140). Interestingly, 14 out of these patients (13.86%) had a presumptive diagnosis of GIST, and the diagnosis of GD was only done at the time of laparotomy and pathologic evaluation of the resection specimen.

For patients who required conventional diagnostic investigation, CT was the most frequently used (41.06%, 131 out of 319). Other investigation, including prenatal ultrasound, abdominal ultrasound, upper gastrointestinal series, MRI, endoscopy, EUS, EUS guide fine needle aspiration (EUS-FNA) and Tc-99m were used upon physician’s choice. In pediatric patients, 16.76% (29 out of 173) were found by prenatal ultrasound.

Most patients (94.29%, 264 out of 280) in our study underwent surgery and the others received conservative treatment. Surgical treatments included local resection (70.83%, 187 out of 264), partial gastrectomy (24.62%, 65 out of 264), radical resection (3.79%, 10 out of 264) and local resection plus radical resection (0.76%, 2 out of 264). Endoscopic resection (including EMR and ESD) was performed in 3.41% (9 out of 264) of all the surgical patients.

All included patients were confirmed by pathological examination as GD. However, there were 14 patients (about 4.39%) were suspected as cancerous. Furthermore, all the 14 patients are adults and 4 out of them accompanied with the rising of tumor markers. Of the 14 GD with malignant transformation, ten were adenocarcinoma [13–22], two were mixed adenocarcinoma and squamous cell carcinoma [23, 24], one was carcinoid [25] and one was neuroendocrine carcinoma [26]. Of the 14 patients, local resection was performed in two patients, radical resection was performed in ten patients, and local resection plus radical resection was performed in two patients. Among them, one patient underwent neoadjuvant treatment and five patients underwent adjuvant therapy. The outcomes of these patients showed all the GD were uneventful. But 4 out of the 14 cancerous patients (28.57%) developed multiple metastases, pelvic metastases, hepatic metastases and peritoneal metastases respectively at postoperative of 6 months, 14 months, seven months and seven months. In addition, one patient of the cancerous patients developed local recurrence and multiple liver metastases in the eighth postoperative month and died at the 14^th^ month postoperatively.

In this study, 77 (24.14%) of cases had reported the follow-up information, and the follow-up period ranges from one month to ten years. The outcomes showed that all patients who were considered as GD without malignant transformation were uneventful.

Consistent with previous studies [2, 27–31], our study also revealed: (a) most GD are cystic in nature, with 87.04% of lesions being cystic in our study; (b) the greater curvature of stomach appears to be the most common site for GD, accounting for 53.30% of the lesions identified in our studies; and (c) pancreas anomaly (especially ectopic pancreas) is the most common congenital malformation (52.94%, 45 out of 85) among all the combined malformations which associated with GD.

## Discussion

GD represent 2–8% of all alimentary tract duplications, with an incidence of 17/1,000,000 in general population [8, 9]. The precise pathogenesis of GD is still unclear but several theories have been proposed including split notochord, persistent embryological diverticula, McLetchie's theory, phylogenetic reversion, aberrant luminal canalization and intrauterine trauma [32–38]. All these theories may explain some of the duplications of the alimentary tract. But there is no single theory can completely explain the formations of gastrointestinal tract duplications in this area. The essential criteria for the diagnosis of GD have been summarized by Rowling [39]: (a) the wall of the cyst is contiguous with the stomach wall; (b) the cyst is surrounded by smooth muscle, which is continuous with the gastric muscle; and (c) the cyst is lined by alimentary epithelium, which is not always gastric type, may be colonic, jejunal and any other type of gut mucosa. GD usually shares a common wall with the stomach and has a common blood supply, which is in contrast to diverticulum.

Although there are some reports claiming that GD are more common in males [4, 40], some authors find that female patient suffers more [28, 31, 41, 42]. While in our study, the results showed that there was no difference in gender distribution, which was similar with the previous system review reported by Aodhnait S. Fahy in 2019 [36]. As is known to all, GD is a congenital disease. Although there is no consensus on its exact aetiology, it seems that none of all these hypotheses suggest that the gender or hormone might play an important role in the development of GD. Therefore, combined with our study results, we hypothesized that there might be some selection bias in previous studies, so they found that this disease was more common in men or women.

The signs and symptoms depend on the size of GD and the age of onset. Clinical findings in patients with duplications include recurrent abdominal pain, vomiting, melena, abdominal distension, recurrent pancreatitis, weight loss, gastric outlet obstruction and failure to thrive, which is similar with the previous studies [1, 27]. These symptoms may be caused by mucosal ulceration, increased pressure of GD cyst and pancreatic ducts involvement [43, 44]. Symptoms of abdominal pain, weight loss and abdominal distension were seen more frequently in older patients, while vomiting and gastrointestinal hemorrhage were more common in pediatric patients. However, none of these symptoms are specific for GD. In our study, the largest GD was found in a 13-year-old girl with the longest diameter of 40 cm on the greater curvature of the stomach, which was reported by M. Davenport in 1999 [43]. The symptom of this girl was gradual abdominal distention without abdominal pain and vomiting, and the lesion was successfully resected with uneventful recovery. In addition, about 26.28% (36 out of 137) adults GD patients and 18.87% (30 out of 159) pediatric patients were asymptomatic, most of them were found during physical examination or prenatal ultrasound.

Due to the lack of specific symptoms and signs, preoperative diagnosis of duplication cyst is very difficult, and it occasionally may mimic GIST, neoplasms and other diseases in clinical presentation and imaging studies. Definitive diagnosis of GD is based on surgical observation and pathologic analysis of the resected specimens. In the current study, 64.89% (207 out of 319) of patients were misclassified with the other gastric tumors, and the diagnosis of GD was certain in only 35.11% (112 out of 319) of patients. Thus, it is necessary to effectively make the correct diagnosis with the help of effective auxiliary examination. Multiple imaging modalities are helpful to identify GD. In this study, preoperative diagnostic studies, including CT, abdominal ultrasound, prenatal ultrasound, MRI, EUS, EUS-FNA, upper gastrointestinal series and Tc-99 m, were useful in making the preoperative diagnosis.

CT is the most frequent choice to image duplication cysts. In spite of advances in imaging, some studies showed that CT scan still misclassify foregut duplication cysts as soft tissue masses in 43% of patients [45]. EUS has been reported to be able to identify the structure of the gastric wall and to identify which layer of the gastric wall the lesion originated from. Thus, EUS has been considered to be the best diagnostic method for GD [9, 46, 47]. Although EUS has these advantages, misdiagnosis still has been reported [48]. In our study, EUS was performed in 14.73% (47 out of 319) of these patients, and only 33.3% of the patients was considered to be diagnosed of GD preoperatively. Compared with the overall patients in our study, EUS did not show its superiority in the diagnosis of GD.

Ultrasound may be helpful in the diagnosis of GD. In the ultrasonography, GD are visible as anechoic cysts [49], unless they show ulceration or bleeding [50]. The wall of the cyst may show an inner hyperechoic layer representing the mucosa and a hypoechoic outer layer representing the muscular layer [51]. Abdominal ultrasound may be helpful to the diagnosis of GD. In addition, with the application of prenatal ultrasound, prenatal diagnosis frequency is increasing [52]. The importance of prenatal diagnosis of GD has been emphasized by several authors [53–55]. Accurate prenatal diagnosis may avoid life-threatening complications. In our study, 16.76% (29 out of 173) of pediatric patients were found by prenatal ultrasound. We suggest that attention should be paid to the presence of GD during prenatal examination.

The role of fine needle aspiration in GD remains controversial. Some surgeons suggested that a cytological and histological examination of gastric duplications via EUS-FNA was necessary to rule out malignancy [9, 56–58]. The previous study confirmed that EUS-FNA was safe and accurate in the diagnosis of foregut duplication cysts [45]. In our study, 15 (4.70%) patients underwent EUS-FNA, and the pathology results were mostly suggestive of inflammatory infiltration, mucus and normal gastric mucosa. Interestingly, 4 out of the 16 patients (25%) has elevated CEA or CA199 levels in the intracapsular liquid without evidence of cancerous of GD. We speculated that the elevated tumor markers had the potential to be associated with malignant transformation of GD in the further.

In addition, Tc-99 m-pertechnetate scintigraph has been reported as a useful modality for preoperative localization of the ectopic functioning gastric mucosa in the GD with sensitivity of 75–85% [59–61]. But at present, this test is mostly used in thyroid diseases, and rarely used in the diagnosis of GD. In our study, there were eight out of the included 319 patients (2.51%) were ordered Tc-99 m-pertechnetate scintigraphy. Recently, Masahiro Kitami has introduced a dynamic compression as a new diagnostic technique [62]. This new technique may enable radiologists to accurately differentiate enteric duplication from other abdominal cysts. However, the population included in our study seems to have heterogeneous demographic characteristics and clinical features, which may represent a problem in the evaluation of the best diagnostic tools and of the most appropriate therapeutic choices for each category of patients. Thus, although multiple imaging modalities are available to identify GD currently, but the optimal method for the diagnosis of GD is still lacking. In clinical practice, it is wise to combine different auxiliary examinations together to confirm the diagnosis of GD and to reduce misdiagnosis.

Malignant transformation should be suspected if any abnormal solid component is found within the GD or serum CEA or CA199 level is elevated. Unfortunately the previous studies showed that the prognosis of GD with malignant transformation was poor despite radical surgery [40], which was similar to our findings.

The gastric duplications can lead to severe adverse events, such as obstruction, torsion, perforation, hemorrhage, and moreover the malignant transformation [14, 23, 48, 63]. Gastric duplication cysts with malignancy carry poor prognosis. Thus, surgical resection must be strongly suggested once the disease is confirmed. The surgical treatment must be chosen depending on its type, for cystic type of GD complete resection is mandatory. While the tubular type of duplications usually not require any intervention when both gastric lumens are patent [60]. We recommend that gastric duplication cysts should be excised whenever detected especially in adults even in asymptomatic cases. GD resection is the treatment of choice, but for those who with a common wall and dual blood supply may mean resection of a portion of the normal stomach [43]. Currently, there are no criteria to guide the choice of surgical strategies. The surgical strategy was determined by the surgeon according to his own experience and the status of patients.

In our study, surgical intervention was performed in the vast majority of patients, and conservative management was performed in only 5.71% (16 out of 280) of patients. The conservative strategies included H2-receptor antagonist [64], antacids and gastrokinetics [65], triplex *Helicobacter pylori* (HP) eradication therapy and PPI maintenance therapy [66], or follow up without medication [67]. Interestingly, we were surprised to find that all of these conservative patients were tubular type of GD, and their outcomes were uneventful. Thus, for tubular type of GD, conservative treatment maybe an optional treatment strategy. The conservative treatment regimen is decided by the respective attending physician. The patients should be followed up closely and regularly as GD has the potential of malignancy.

In conclusion, in order to improve the accurate preoperative diagnosis rate and offer advice on treatment and management of GD, we recommend the following items: How to improve the accurate preoperative diagnosis of GD: (1) Prenatal ultrasound is necessary for prenatal diagnosis. (2) CT, EUS (with or without FNA), abdominal ultrasound and upper gastrointestinal series may helpful for the diagnosis of GD. (3) if condition permit, Tc-99 m-pertechnetate scintigraph can be considered. (4) Blood test (including CEA, CA199, CA72-4) is recommended as a routine test. How to select the appropriate treatment strategy for GD: (1) Cystic type of GD should be excised whenever detected especially in adults even in asymptomatic cases. The surgical approach should be determined by the surgeon’s experience, the GD’s feature and the status of patients. (2) For tubular type of GD, conservative treatment maybe an optional treatment strategy, and the treatment regimen is decided by the physician. During conservative treatment the patients should be followed up closely and regularly to prevent complications and malignant transformation. (3) When the malignant transformation evidence of GD is found, radical resection is recommended. GD with malignancy carry poor prognosis.

## Conclusions

Different from what has been reported in the previous studies, we found that there is no significant difference in gender distribution in the onset of GD. In addition, consistent with previous studies, we also found: (a) the greater curvature is the most common site of GD; (b) the cystic GD is the most common type of GD; and (c) pancreas anomaly is the most common congenital malformation associated with GD. Gastric duplications can present with a variety of symptoms. For relieving symptoms and preventing malignant change, all GD should be considered for surgery. But the surgical approach should be individualized. For cystic gastric duplications, optimal treatment is completely surgical removal. While for tubular gastric duplications, conservative treatment maybe an alternative strategy under closely and regularly follow up.

To our best knowledge, this is the largest sample size pooled analysis of GD. However, the results still need to be interpreted with caution for some data had not been described previously and especially there currently lack long-term follow-up data of survival time.

## Data Availability

All relevant data used in this study have been included in the manuscript. The corresponding author can be contacted if any further information is needed.

## Abbreviations

GD: Gastric duplications
GIST: Gastrointestinal stromal tumor
F: Female
M: Male
CT: Computed tomography
MRI: Magnetic resonance imaging
EUS: Endoscopic ultrasonography
EUS-FNA: Endoscopic ultrasonography guide fine needle aspiration
ESD: Endoscopic submucosal dissection
EMR: Endoscopic mucosal resection
PKUPH: Peking University People’s Hospital
SD: Standard deviation
AW: Anterior wall of stomach
PW: Posterior wall of stomach
ANED: Alive with no evidence of disease
AWD: Alive with disease
DOD: Dead of disease
